# The CUBED Versus ABCDE Recognition Tools in the Detection of Malignant Melanomas Arising in the Foot: An Analysis of Four Recent Case Studies in a Single NHS Community Trust

**DOI:** 10.1002/jfa2.70159

**Published:** 2026-04-29

**Authors:** Amy Sherratt, Emily Vaughan, Nekita Huckerby, Hannah Winn, Margaret Swithenbank

**Affiliations:** ^1^ Derbyshire Community Health Services Derby UK; ^2^ University Hospitals of Derby and Burton NHS Foundation Trust Derby UK

**Keywords:** ABCDE, CUBED, dermatology, foot, melanoma, podiatry

## Abstract

**Background:**

Melanoma of the foot and nail unit carries a worse prognosis than cutaneous melanoma elsewhere because lesions are frequently hidden, misdiagnosed, and therefore diagnosed at more advanced stages. Traditional ABCDE criteria are often insufficient for foot and nail presentations; the CUBED recognition tool was developed to improve detection of atypical plantar and subungual lesions.

**Aim:**

To evaluate four consecutive cases of foot melanoma seen in a single NHS community podiatry department, compare case characteristics against ABCDE and CUBED recognition tools, and describe service responses that aimed to improve early detection.

**Methods:**

A retrospective case series of four patients diagnosed with foot melanoma between 2019 and 2022 in one community podiatry service. Clinical features, referral timelines, diagnostic pathways, treatments, and outcomes were extracted and mapped against ABCDE and CUBED criteria. Shared‐learning interventions developed by the department following these cases are described.

**Results:**

All four cases were toe‐based melanomas (one lentigo maligna, one nail‐bed malignant melanoma, two acral melanomas). Presentations commonly included irregular colour, bleeding or ulceration, delayed healing, diagnostic uncertainty, and lesion enlargement. CUBED criteria more consistently identified concerning features across the series than ABCDE criteria, prompting urgent dermatology referral in each case. Outcomes varied: one patient remains disease‐free after excision, one developed distant metastasis and died, two underwent toe amputation and received systemic treatment. Departmental responses included case‐based teaching and a poster campaign promoting CUBED and ABCDE recognition across local clinical networks.

**Conclusion:**

Podiatrists are pivotal in early detection of foot melanoma. The CUBED tool demonstrated greater sensitivity for atypical foot and nail lesions in this series. Wider education and adoption of CUBED alongside ABCDE may reduce misdiagnosis, shorten diagnostic delay, and improve clinical outcomes.

## 1 Introduction

Melanoma is a malignant tumour arising from melanocytes [1]. It is the fifth most common cancer in the UK and accounts for more deaths than all other skin cancers combined [2]. Global incidence rates of melanoma skin cancer rose by 44% between 2008 and 2018 with deaths increasing by 32% [3]. Around 2%–3% of all cutaneous melanoma occur on the foot [4]. With an average Mortality‐to‐Incidence Ratio of 21%, melanoma skin cancer remains one of the most treatable cancers [3]. However, delaying treatment of a stage 1 melanoma by just 1 month increases the risk of death by 5% [5].

Acral Lentiginous Melanomas arise on the foot, hands and nail unit, particularly in people with darker skin tones, and hold a poorer prognosis than cutaneous melanoma elsewhere in the body [6]. This has been attributed to numerous factors; melanomas of the feet are frequently concealed in interdigital spaces, under nail plates or on the plantar surface, making them harder to see and to self‐detect [7]. Further still, it has been suggested that 25%–66% of lesions are regularly misdiagnosed as other common foot disorders such as ingrowing toenails, ulceration, and necrosis [8]. Delay in presentation to dermatology and subsequent diagnosis can result in thicker more advanced tumours than if they had been detected earlier [7].

**FIGURE 1:**
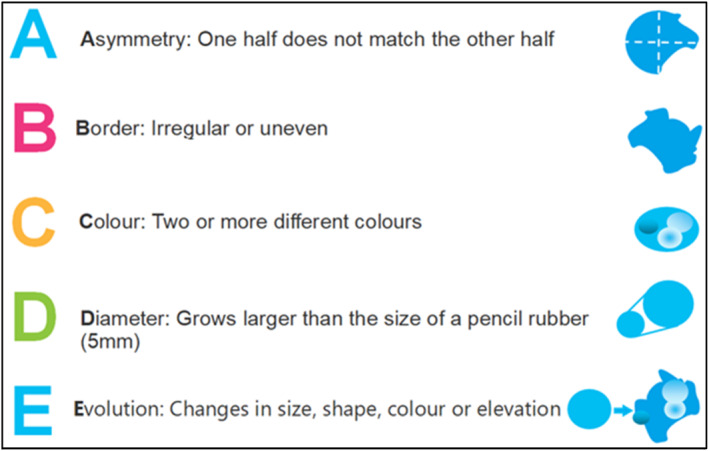
ABCDE recognition tool for detecting melanomas.

Historically the ABCDE acronym (Figure 1.) has been used to evaluate pigmented skin lesions on all body sites, particularly on sun exposed areas, and prompt onward referral for expert opinion if any of its characteristics are met [9]. However, Bristow and Acland [10], suggested it is not well suited for analysing lesions on none sun exposed area such as the nail unit and plantar surface due to the large variation in presentations of melanomas at these sites.

**FIGURE 2:**
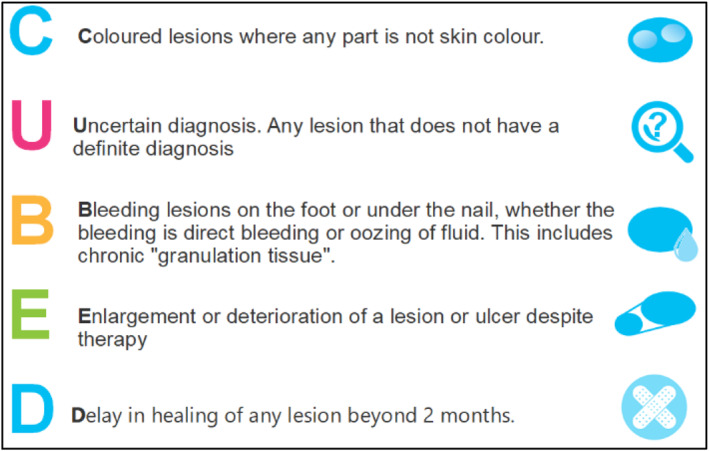
CUBED recognition tool for detecting melanomas of the foot and nail unit.

In response to both the literary and clinical evidence and utilising an expert review panel, Bristow and de Berker [8], created an alternative set of assessment criteria under the acronym of ‘CUBED’ (Figure 2), for the assessment of pigmented lesions specifically occurring on the foot and nail unit. The tool aims to increase the likelihood of earlier detection and subsequent onward referral through identifying lesions that are ‘unusual’ in form with atypical features that might not be as easily identified with the ABCDE acronym. If two or more CUBED characteristics are identified, then the patient should be referred urgently for specialist dermatological assessment to aid prompt diagnosis and treatment [8].

## 2 Methodology

**FIGURE 3:**
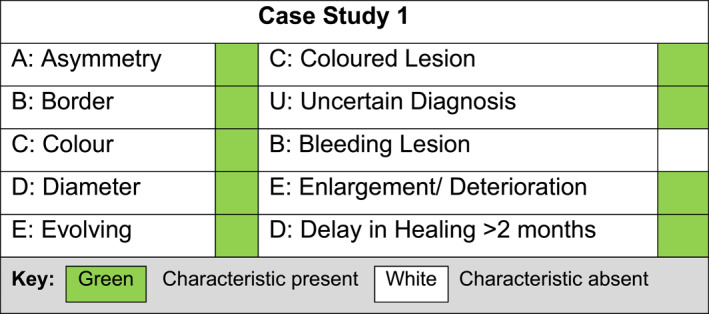
Podiatrists comparison of clinical signs against the ABCDE and CUBED recognition tools for case study 1.

**FIGURE 4:**
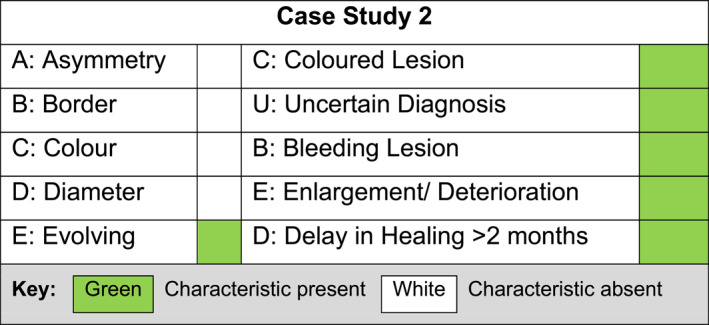
Podiatrists comparison of clinical signs against the ABCDE and CUBED recognition tools for case study 2.

**FIGURE 5:**
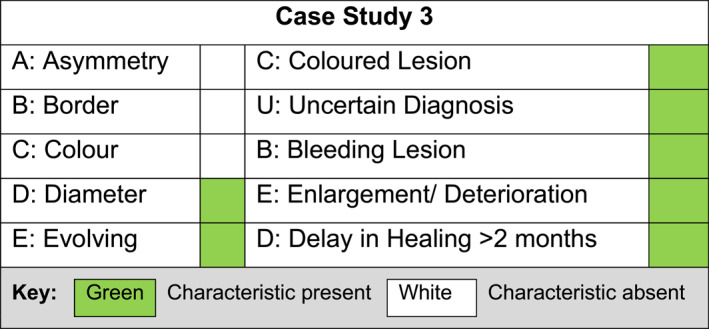
Podiatrists comparison of clinical signs against the ABCDE and CUBED recognition tools for case study 3.

**FIGURE 6:**
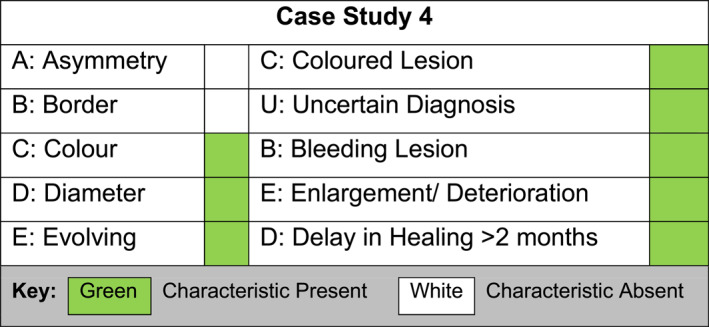
Podiatrists comparison of clinical signs against the ABCDE and CUBED recognition tools for case study 4.

Four recent patient cases within a single NHS community podiatry department in the east midlands region of England where malignant melanoma on the foot was diagnosed were identified. Clinicians felt this could provide a unique opportunity for shared learning and service development as a case series review. Each case was compared and analysed against both the ABCDE and CUBED recognition tools collectively by the four podiatrists that described them, to help tailor departmental and trust‐wide training for recognising such lesions. The four case studies are detailed below and their clinical characteristics plotted against the two recognition tools in Figures 3, 4, 5, 6.

## 3 Case Studies

### 3.1 Case Study 1, Male, Aged 83, Left 1st Plantar Toe, Lentigo Maligna

**FIGURE 7:**
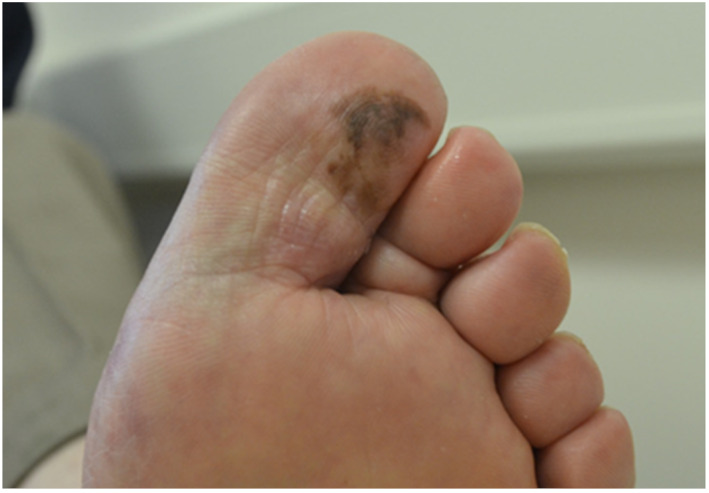
Left first plantar toe lentigo maligna.

An 83‐year‐old male presented with a 20 mm brown irregularly pigmented patch under the Left 1st toe (Figure 7). The area had been noted by a nurse 2 months prior with an uncertain diagnosis and was enlarging. The lesion was asymptomatic, with no itching, bleeding or elevation. The lesion was asymmetrical in colour and had a border with a diameter above 5 mm. An urgent request for a dermatology referral was sent to the GP and a Dermatology review and biopsy was performed within 3 weeks. Histology showed a diagnosis of Lentigo maligna and the patient subsequently had a wide local excision with grafting. A few months post operatively the same podiatrist noticed a slight discolouration to the scar site and referred back to plastic surgery, the melanoma had reoccurred requiring deeper excision but fortunately it had not spread further. The patient has now moved on to 6 monthly reviews with dermatology and remains well and cancer free.

### 3.2 Case Study 2, Female, Aged 85, Right 5th Nail Bed, Malignant Melanoma

**FIGURE 8:**
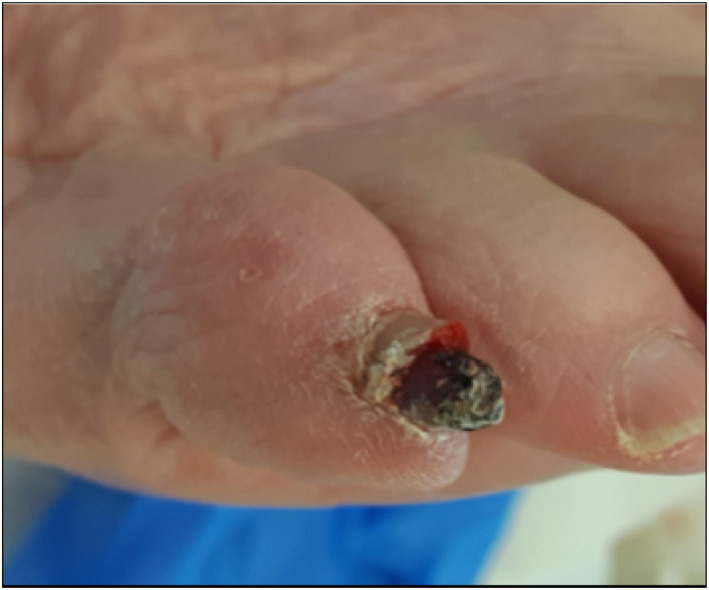
Right 5th nail bed malignant melanoma.

An 85‐year‐old female patient was referred into Podiatry with a 10 months history of a lesion on the right little toe (Figure 8). The lesion was assessed as having a colour change, an uncertain diagnosis, bleeding, continued enlargement, and a delay in healing beyond 2 months. An urgent task was sent to the GP and an urgent referral to Dermatology was made. Excision of the lesion was carried out by a Dermatologist 9 days later. The lesion was confirmed as a malignant melanoma and the toe was amputated 32 days later and the patient underwent systemic treatment.

### 3.3 Case Study 3, Male, Aged 73, Right 1st Subungual, Acral Lentigous Malignant Melanoma

**FIGURE 9:**
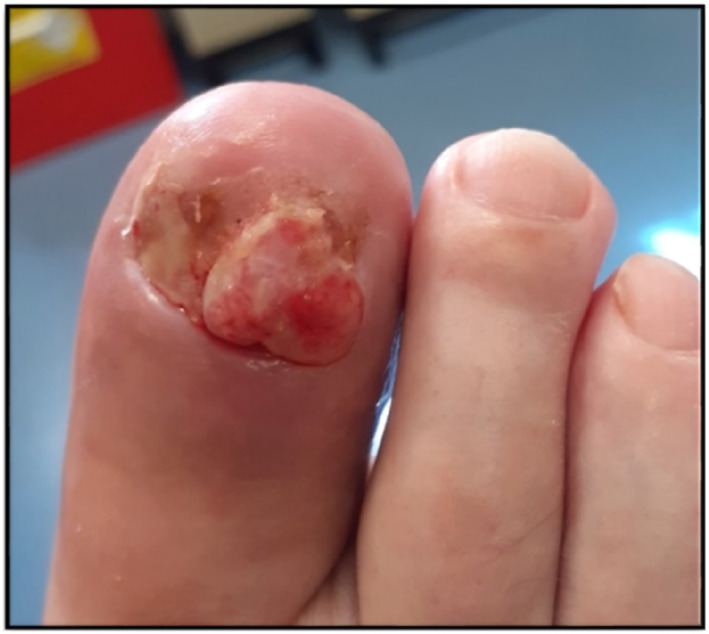
Right 1st subungual acral lentigous malignant melanoma.

A 73‐year‐old gentleman presented with a 4 weeks history of an ingrowing toenail and hypergranulation tissue. The lesion had irregular borders, was bleeding and had enlarged recently. Nail surgery was performed within 2 weeks for suspected hypergranulation associated with ingrowing nails. After nail removal the lesion continued to grow (Figure 9). The diagnosis became uncertain, and there was a delay in healing despite treatment. The patient was referred to the sarcoma unit and subsequently dermatology, who initially performed a wide local excision, with a diagnosis of acral lentiginous malignant melanoma with a subsequent hallux amputation. Sadly for this gentleman his cancer had metastasised and he died 18 months following initial presentation.

### 3.4 Case Study 4, Male, Aged 63, Left 3rd Nail Bed, Acral Malignant Melanoma

**FIGURE 10:**
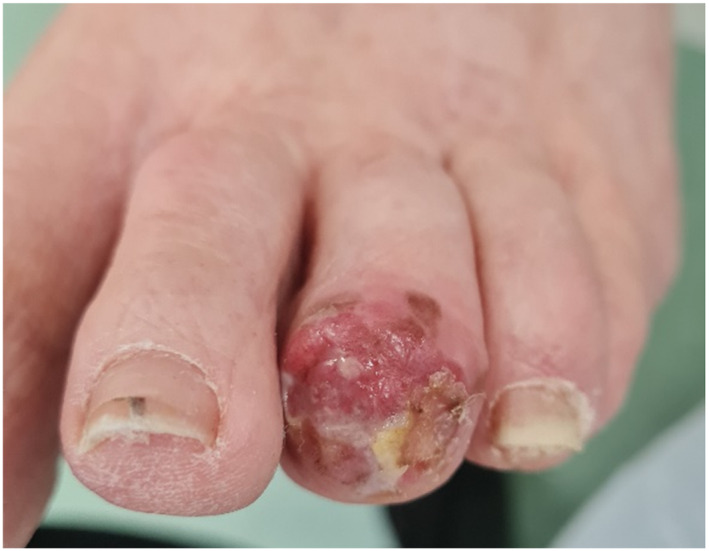
Left 3rd nail bed acral malignant melanoma.

A 63‐year‐old gentleman was referred to Podiatry with a slow healing wound to his left 3rd nail bed following 6 months of input from the practice nurse (Figure 10). On examination there was a fungating lesion with ulceration, irregular colour, an uncertain diagnosis, bleeding, and enlargement of the wound over time. The patient was referred for an urgent dermatology assessment. Following a biopsy, acral malignant melanoma was diagnosed. Shortly after this diagnosis the toe was amputated and a sentinel lymph node biopsy was arranged. Three sentinel lymph nodes were excised, one of which was positive, indicating metastatic melanoma. The patient started immunotherapy and remains under the care of oncology for ongoing cancer treatment.

## 4 Discussion

The use of the CUBED recognition tool within the four case studies presented above helped to aid diagnosis and prompt urgent onward referral to specialist services. The traditional ABCDE analogy might not have provoked such action particularly in case studies 2 to 4. These findings mirror the recommendations by Bristow and de Berker [8], who suggest the CUBED recognition tool is sensitive at detecting melanoma of the foot.

All four of the case studies were examples of melanomas of the toe. The patients in case studies 2–4 had bleeding or ulcerated lesions which is associated with more advanced disease and poor 5 years survival rates in the literature [11].

In every case study the patients were referred to the podiatrist with their lesions as their primary presenting complaint. This reinforces the suggestion that podiatrists play a vital role in the screening and detection of foot and lower limb melanomas [8]. This highlights the importance that the profession requires a good understanding of how to recognise melanomas to fulfil their role appropriately.

**FIGURE 11:**
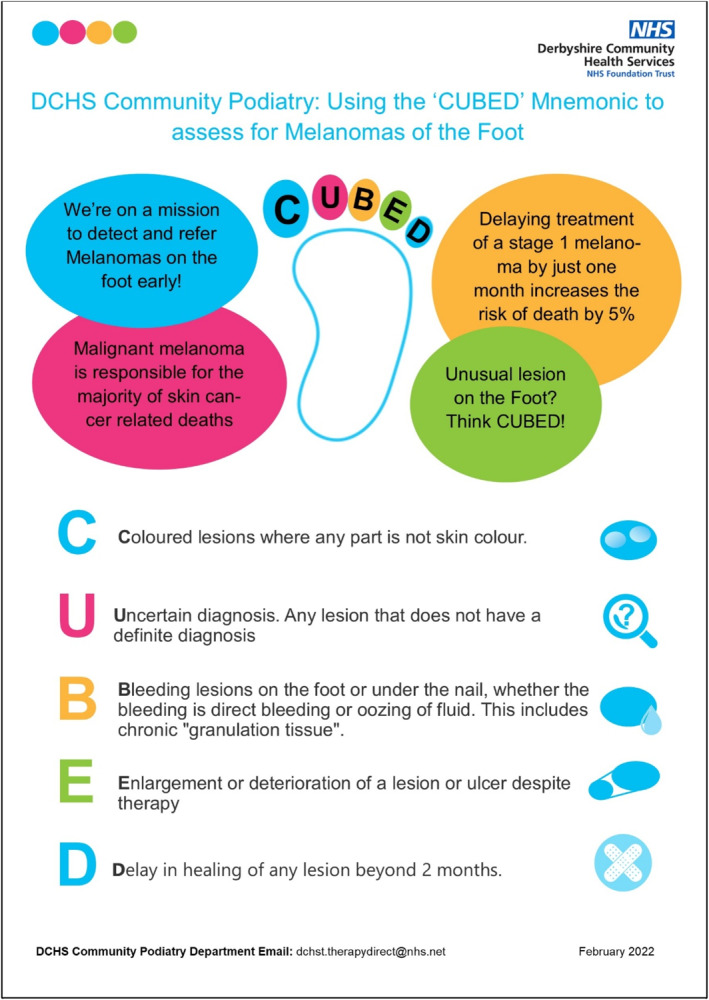
CUBED awareness poster used for the CUBED awareness campaign.

Following each case the podiatrist, with the consent of the patient, presented the clinical history and findings to their teams for shared learning. Four cases occurred in one department in less than 3 years leading the podiatrists to develop a poster campaign highlighting the CUBED and ABCDE recognition tools and their importance in detecting melanomas of the feet (Figure 11).

The poster and the case studies were shared across local meetings, local wound improvement webinars and allied health professionals forums to ensure awareness was raised across a variety of professions involved in the care of patients with foot problems.

## 5 Limitations

This article has several limitations including its small sample size of 4 case studies taken from a single NHS trust which inhibits the generalisability of the findings. The retrospective and descriptive nature of the case reports also lends the possibility of author bias whilst not allowing for strong statistical evaluation of the recognition tools that it assessed. A large‐scale, multi‐centre study comparing the ABCDE and CUBED recognition tools to determine their level of specificity in detecting melanomas of the foot would help assess this area further and provide evidence for robust clinical guidelines.

## 6 Conclusion

Podiatrists are at the forefront of treating foot disease and need to have a high suspicion for detecting melanomas of the foot to ensure early diagnosis and better prognosis for their patients. More work needs to be done to raise awareness of the CUBED recognition tool amongst podiatrists and other health care professionals. Increased awareness may reduce the current high rates of misdiagnosis and poor clinical outcomes commonly seen in melanoma of the foot.

## Author Contributions

**Amy Sherratt:** conceptualization, data curation, formal analysis, investigation, methodology, project administration, resources, validation, visualization, writing – original draft, writing – review and editing. **Emily Vaughan:** validation, visualization, writing – original draft, writing – review and editing. **Nekita Huckerby:** conceptualization, data curation, formal analysis, investigation, resources, validation, visualization, writing – original draft, writing – review and editing. **Hannah Winn:** conceptualization, data curation, formal analysis, investigation, resources, validation, visualization, writing – original draft, writing – review and editing. **Margaret Swithenbank:** conceptualization, data curation, formal analysis, investigation, resources, validation, visualization, writing – original draft, writing – review and editing.

## Funding

No funding was received in order to produce this article. The authors would like to thank the Royal College of Podiatry who provided funding to cover the publication fees of this article in the Journal of Foot and Ankle Research. The authors would also like to thank Derbyshire Community Health Services and the University Hospitals of Derby and Burton for providing clinical time to work on this piece.

## Ethics Statement

As this is a case review no ethical approval was needed to create this work. Consent was gained from the patients using the trusts standard operating procedure following information governance guidelines.

## Consent

All four patients whose case studies are shown in this article gave full verbal and written consent to the use of their clinical details and images being used for publication and sharing globally.

## Conflicts of Interest

The authors declare no conflicts of interest.

## Permission to Reproduce Material From Other Sources

The authors give permission for the material to be reproduced for educational purposes in line with the patients consent forms.

## Data Availability

As this is a case series review the source data that supports the conclusions is not publicly available to ensure confidentiality of the patients involved.
